# Supplementary material for: Isolation and identification of microorganisms associated with automated teller machines on Federal Polytechnic Ede campus
Source: PLoS One. 2021 Aug 5;16(8):e0254658. doi: 10.1371/journal.pone.0254658 (PMC8341644; doi:10.1371/journal.pone.0254658)
Supplement: S1 Table — (DOCX) [file pone.0254658.s001.docx]

**S1 Table: Observed Bacterial Growth from Inoculating Swab Sticks (Mixed Culture)**

| S/N | CODE ISOLATE | MEDIUM | GROWTH | COLOUR | SHAPE |
| --- | --- | --- | --- | --- | --- |
| 1 | 1A^ONE^ | NA | + | CREAM | ROUND |
| 2 | 1A^TWO^ | NA | + | CREAM | ROUND |
|  |  |  |  | ORANGE | ROUND |
| 3 | 1B^ONE^ | NA | + | CREAM | ROUND |
| 4 | 1B^TWO^ | NA | + | CREAM | ROUND |
|  |  |  |  | CREAM | FLAT |
| 5 | 2A^ONE^ | NA | + | CREAM | ROUND |
| 6 | 2A^TWO^ | NA | + | CREAM | ROUND |
| 7 | 2B^ONE^ | NA | + | CREAM | ROUND |
| 8 | 2B^TWO^ | NA | + | ORANGE | ROUND |
|  |  |  |  | CREAM | ROUND |
| 9 | 3A^ONE^ | NA | + | CREAM | ROUND |
| 10 | 3A^TWO^ | NA | + | CREAM | ROUND |
| 11 | 3B^ONE^ | NA | + | ORANGE | FLAT |
|  |  |  |  | CREAM | ROUND |
| 12 | 3B^TWO^ | NA | + | CREAM | SERATED |
| 13 | 4A^ONE^ | NA | + | CREAM | ROUND |
|  |  |  |  | CREAM | ROUND |
| 14 | 4A^TWO^ | NA | + | CREAM | ROUND |
| 15 | 4B^ONE^ | NA | + | CREAM | SERATED |
|  |  |  |  | CREAM | ROUND |
| 16 | 4B^TWO^ | NA | + | CREAM | ROUND |
| 17 | 5A^ONE^ | NA | + | CREAM | FLAT |
|  |  |  |  | CREAM | ROUND |
| 18 | 5A^TWO^ | NA | + | CREAM | SERATED |
| 19 | 5B^ONE^ | NA | + | CREAM | ROUND |
| 20 | 5B^TWO^ | NA | + | CREAM | SERATED |
| 21 | 6A^ONE^ | NA | + | CREAM | ROUND |
| 22 | 6A^TWO^ | NA | + | ORANGE | FLAT |
| 23 | 6B^ONE^ | NA | + | CREAM | ROUND |
|  |  |  |  | ORANGE | ROUND |
| 24 | 6B^TWO^ | NA | + | CREAM | ROUND |
| 25 | 7A^ONE^ | NA | + | CREAM | ROUND |
|  |  |  |  | ORANGE | FLAT |
| 26 | 7A^TWO^ | NA | + | CREAM | ROUND |
| 27 | 7B^ONE^ | NA | + | CREAM | FLAT |
|  |  |  |  | CREAM | ROUND |
| 28 | 7B^TWO^ | NA | + | ORANGE | ROUND |
| 29 | CONTROL | NA | - | - | - |

S1 Table shows the observed growth of the mixed culture that is the original isolates that were collected from the ATM machines themselves. There was growth on almost all the plates except controls, and the color on the plates were mainly cream and orange, while the shapes on the observed plates were mostly round, flat, and serrated.
