# Supplementary material for: Isolation and identification of microorganisms associated with automated teller machines on Federal Polytechnic Ede campus
Source: PLoS One. 2021 Aug 5;16(8):e0254658. doi: 10.1371/journal.pone.0254658 (PMC8341644; doi:10.1371/journal.pone.0254658)
Supplement: S2 Table — (DOCX) [file pone.0254658.s002.docx]

**S2 Table: Observation of Mixed Culture, Sub-Cultured Onto another Plate**

| S/N | CODE ISOLATE | MEDIUM | GROWTH | COLOUR | SHAPE |
| --- | --- | --- | --- | --- | --- |
| 1 | 1A^ONE^ | NA | + | CREAM | ROUND |
| 2 | 1A^TWO^ | NA | + | CREAM | ROUND |
| 3 | 1A^TWO^ | NA | + | ORANGE | ROUND |
| 4 | 1B^ONE^ | NA | + | CREAM | SERATED |
|  |  |  |  | CREAM | ROUND |
| 5 | 1B^TWO^ | NA | + | CREAM | FLAT |
| 6 | 1B^TWO^ | NA | + | CREAM | ROUND |
| 7 | 1B^TWO^ |  |  | ORANGE | ROUND |
|  |  |  |  | CREAM | SERATED |
| 8 | 2A^ONE^ | NA | + | CREAM | ROUND |
|  |  |  |  | CREAM | SERATED |
| 9 | 2A^TWO^ | NA | + | CREAM | ROUND |
| 10 | 2B^ONE^ | NA | + | CREAM | ROUND |
| 11 | 2B^TWO^ | NA | + | ORANGE | ROUND |
| 12 | 2B^TWO^ | NA | + | CREAM | ROUND |
| 13 | 3A^ONE^ | NA | + | CREAM | ROUND |
| 14 | 3A^TWO^ | NA | + | CREAM | SERATED |
|  |  |  |  | CREAM | ROUND |
| 15 | 3B^ONE^ | NA | + | ORANGE | ROUND |
|  |  |  |  | CREAM | SERATED |
| 16 | 3B^ONE^ | NA | + | CREAM | ROUND |
| 17 | 3B^TWO^ | NA | + | ORANGE | ROUND |
|  |  |  |  | CREAM | SERATED |
| 18 | 3B^TWO^ | NA | + | CREAM | FLAT |
| 19 | 4A^ONE^ | NA | + | CREAM | ROUND |
|  |  |  |  | CREAM | FLAT |
| 20 | 4A^TWO^ | NA | + | CREAM | ROUND |
| 21 | 4B^ONE^ | NA | + | CREAM | SERATED |
| 22 | 4B^ONE^ | NA | + | CREAM | SERATED |
|  |  |  |  | CREAM | ROUND |
| 23 | 4B^TWO^ | NA | + | CREAM | ROUND |
| 24 | 5A^ONE^ | NA | + | CREAM | SERATED |
|  |  |  |  | CREAM | ROUND |
| 25 | 5A^TWO^ | NA | + | CREAM | FLAT |
| 26 | 5B^ONE^ | NA | + | CREAM | ROUND |
| 27 | 5B^TWO^ | NA | + | CREAM | SERATED |
| 28 | 6A^ONE^ | NA | + | CREAM | ROUND |
| 29 | 6A^TWO^ | NA | + | ORANGE | ROUND |
| 30 | 6B^ONE^ | NA | ­ + | CREAM | ROUND |
| 31 | 6B^ONE^ | NA | + | CREAM | SERATED |
| 32 | 6B^TWO^ | NA | + | CREAM | ROUND |
| 33 | 7A^ONE^ | NA | + | CREAM | SERATED |
| 34 | 7A^ONE^ | NA | + | CREAM | ROUND |
| 35 | 7A^TWO^ | NA | + | CREAM | SERATED |
| 36 | 7B^ONE^ | NA | + | CREAM | ROUND |
| 37 | 7B^ONE^ | NA | + | CREAM | SERATED |
| 38 | 7B^TWO^ | NA | + | CREAM | ROUND |
| 39 | CONTROL | NA | - | - | - |

S2 Table shows the second phase of culturing, where the original isolates were sub cultured into another set of plates. This time, it was observed that the colour of the colonies were mainly cream and few were orange, while there was almost an even distribution between the serrated and round shapes with few flat ones.
