# Supplementary material for: Isolation and identification of microorganisms associated with automated teller machines on Federal Polytechnic Ede campus
Source: PLoS One. 2021 Aug 5;16(8):e0254658. doi: 10.1371/journal.pone.0254658 (PMC8341644; doi:10.1371/journal.pone.0254658)
Supplement: S3 Table — (DOCX) [file pone.0254658.s003.docx]

**S3 Table: Observed Growth of Pure Culture of the Isolates**

| S/N | CODE ISOLATE | MEDIUM | GROWTH | COLOUR | SHAPE |
| --- | --- | --- | --- | --- | --- |
| 1 | 1A^ONE^ | NA | + | CREAM | ROUND |
| 2 | 1A^TWO^ | NA | + | CREAM | ROUND |
| 3 | 1A^TWO^ | NA | + | ORANGE | FLAT |
| 5 | 1B^ONE^ | NA | + | CREAM | ROUND |
| 6 | 1B^ONE^ | NA | + | CREAM | SERATED |
| 7 | 1B^TWO^ | NA | + | CREAM | ROUND |
| 8 | 1B^TWO^ | NA | + | CREAM | ROUND |
| 9 | 1B^TWO^ | NA | + | CREAM | SERATED |
| 10 | 2A^ONE^ | NA | + | CREAM | ROUND |
| 11 | 2A^ONE^ | NA | + | CREAM | SERATED |
| 12 | 2A^TWO^ | NA | + | CREAM | ROUND |
| 13 | 2B^ONE^ | NA | + | CREAM | ROUND |
| 14 | 2B^TWO^ | NA | + | ORANGE | ROUND |
| 15 | 2B^TWO^ | NA | + | CREAM | ROUND |
| 16 | 3A^ONE^ | NA | + | CREAM | ROUND |
| 17 | 3A^TWO^ | NA | + | CREAM | ROUND |
| 18 | 3A^TWO^ | NA | + | CREAM | SERATED |
| 19 | 3B^ONE^ | NA | + | ORANGE | FLAT |
| 20 | 3B^ONE^ | NA | + | CREAM | ROUND |
| 21 | 3B^TWO^ | NA | + | CREAM | SERATED |
| 22 | 3B^TWO^ | NA | + | CREAM | FLAT |
| 23 | 3B^TWO^ | NA | + | ORANGE | ROUND |
| 24 | 4A^ONE^ | NA | + | CREAM | ROUND |
| 25 | 4A^ONE^ | NA | + | CREAM | ROUND |
| 26 | 4A^TWO^ | NA | + | CREAM | ROUND |
| 27 | 4B^ONE^ | NA | + | CREAM | SERATED |
| 28 | 4B^ONE^ | NA | + | CREAM | ROUND |
| 29 | 4B^ONE^ | NA | + | CREAM | SERATED |
| 30 | 4B^TWO^ | NA | + | CREAM | ROUND |
| 31 | 5A^ONE^ | NA | + | CREAM | FLAT |
| 32 | 5A^ONE^ | NA | + | CREAM | ROUND |
| 33 | 5A^TWO^ | NA | + | CREAM | SERATED |
| 34 | 5B^ONE^ | NA | + | CREAM | ROUND |
| 35 | 5B^TWO^ | NA | + | CREAM | SERATED |
| 36 | 6A^ONE^ | NA | + | CREAM | ROUND |
| 37 | 6A^TWO^ | NA | + | ORANGE | FLAT |
| 38 | 6B^ONE^ | NA | + | CREAM | ROUND |
| 39 | 6B^ONE^ | NA | + | ORANGE | ROUND |
| 40 | 6B^TWO^ | NA | + | CREAM | ROUND |
| 41 | 7A^ONE^ | NA | + | CREAM | ROUND |
| 42 | 7A^ONE^ | NA | + | ORANGE | FLAT |
| 43 | 7A^TWO^ | NA | + | CREAM | ROUND |
| 44 | 7B^ONE^ | NA | + | CREAM | FLAT |
| 45 | 7B^ONE^ | NA | + | CREAM | ROUND |
| 46 | 7B^TWO^ | NA | + | ORANGE | ROUND |
| 47 | CONTROL | NA | - | - | - |

*****A^TWO^= Sample collected in the afternoon from ATM dispensing ₦500.

*A^ONE^= Sample collected in the morning from ATM dispensing₦500.

*B^ONE^= Sample collected from ATM machine that dispenses ₦1000.

*B^TWO^ =Sample collected in the afternoon from ATM dispensing ₦1000.

*NA= Nutrient agar.

***+ =** Growth on media.

*- = No growth on media

S3 Table shows further sub-culturing, this time of identified pure cultures with almost a repeat on what was observed in table 2.

In summary, Tables 1-3 showed the bacteria isolated from the ATM machines at different times, with their shapes and color characteristics.
