# Supplementary material for: Isolation and identification of microorganisms associated with automated teller machines on Federal Polytechnic Ede campus
Source: PLoS One. 2021 Aug 5;16(8):e0254658. doi: 10.1371/journal.pone.0254658 (PMC8341644; doi:10.1371/journal.pone.0254658)
Supplement: S4 Table — (DOCX) [file pone.0254658.s004.docx]

**S4 Table. Gram Staining of the Isolates**

| ORGANISM | 1 | 2 | 3 | 4 | 5 | 6 | 7 | 8 | 9 |
| --- | --- | --- | --- | --- | --- | --- | --- | --- | --- |
| GRAM TEST | **+** | **+** | **-** | **+** | **-** | **+** | **-** | **+** | **-** |
| SHAPE | Slightly curved rod | Cocci |  | Rods | Mucoid colony | Cocci in pairs | Straight rod | Rod |  |

*+ positive, - negative

S4 Table shows the gram staining test for the isolates, with 9 (nine) isolates identified with almost even distribution between the gram positive and gram negative tests.
