# Supplementary material for: Isolation and identification of microorganisms associated with automated teller machines on Federal Polytechnic Ede campus
Source: PLoS One. 2021 Aug 5;16(8):e0254658. doi: 10.1371/journal.pone.0254658 (PMC8341644; doi:10.1371/journal.pone.0254658)
Supplement: S5 Table — (DOCX) [file pone.0254658.s005.docx]

**S5 Table. Biochemical Test for Identification of the Isolates**

| ORGANISM | 1 | 2 | 3 | 4 | 5 | 6 | 7 | 8 | 9 |
| --- | --- | --- | --- | --- | --- | --- | --- | --- | --- |
| CATALASE | + | **+** | **-** | + | + | + | + | + | - |
| SUG. FERMENT. | - | **-** | **-** | + | - | - | + | + | - |
| CITRATE | + | **-** | **-** | - | + | - | + | + | - |
| INDOLE | - | **-** | **-** | + | - | - | - | - | - |
| COAGULASE | - | **+** | **-** | - | + | - | - | - | - |
| UREASE | - | **-** | **-** | - | - | - | - | - | - |
| OXIDASE | + | **-** | **-** | - | + | + | - | - | - |
| ORGANISM | **Pseudomonas** | **Staphylococcus** |  | **E. coli** | **Serratia** | **Micrococcus** | **Salmonella** | **Klebsellia** |  |

*+ = positive, - = negative

S5 Table showed the biochemical tests carried for identification of the microbes in the 9 isolates, with isolate 3 and 9 giving no microbial identity, while 7 distinct microbes were identified from plates 1, 2, 4, 5, 6, 7 and 8.
