# Supplementary material for: Isolation and identification of microorganisms associated with automated teller machines on Federal Polytechnic Ede campus
Source: PLoS One. 2021 Aug 5;16(8):e0254658. doi: 10.1371/journal.pone.0254658 (PMC8341644; doi:10.1371/journal.pone.0254658)
Supplement: Attachment — Submitted filename: Comments.docx [file pone.0254658.s006.docx]

Manuscript Number: PONE-D-21-02655

Submission Title: ISOLATION AND IDENTIFICATION OF MICROORGANISMS ASSOCIATED WITH AUTOMATED TELLER MACHINES ON FEDERAL POLYTECHNIC EDE CAMPUS


Dear Authors,

Thanks for submitting the manuscript. No doubt, this research article is intriguing and informative. Your manuscript has been reviewed but it needs substantial revision. My point-to-point comments are listed below. You are encouraged to carefully revise the manuscript, highlighting the exact changes made.

1. According to my opinion, the introduction section needs more literature survey for better literature review.
2. Describe the need of the current study in the presence of other literature in the introduction as well as in conclusion.
3. There should be space between numerical value and its unit like 20 seconds. There is need to correct this mistake carefully in your manuscript.
4. According to my opinion, result section needs to be revised. First, the result should be clearly elaborated and defined. The table numbers should be mentioned in the text.
5. Sample Collection and Processing section demands revision as author has mentioned in abstract, “Swabs were collected from each ATM screen, buttons, floor, user’s hand, and exposure of plates” (Sample Collection and Processing does not provide any information regarding the places from where sample collect)
6. In my opinion, conclusion must be revised and author has mentioned recommendation in separate heading so there is need to remove recommendation word from conclusion heading.
7. Add the limitations of this paper.
8. Some English revision of the manuscript is needed.
