# Supplementary material for: Isolation and identification of microorganisms associated with automated teller machines on Federal Polytechnic Ede campus
Source: PLoS One. 2021 Aug 5;16(8):e0254658. doi: 10.1371/journal.pone.0254658 (PMC8341644; doi:10.1371/journal.pone.0254658)
Supplement: Attachment — Submitted filename: review of article.docx [file pone.0254658.s007.docx]

Repetition of same Reference in consecutive sentence.

Repetition of sentence in same paragraph.

The transport of swabs from sample site to Laboratory is not clear.

Abbreviation were not used properly

Lack of reference for several techniques which are used.

Not sufficient tests were conducted for different species of bacteria

References are not according to Journal Policy and paragraphing of references are very poor.

**Conclusion:**

The title of project was excellent but there are lots of deficiencies, these should be addressed.
