# Supplementary material for: Isolation and identification of microorganisms associated with automated teller machines on Federal Polytechnic Ede campus
Source: PLoS One. 2021 Aug 5;16(8):e0254658. doi: 10.1371/journal.pone.0254658 (PMC8341644; doi:10.1371/journal.pone.0254658)
Supplement: Attachment — Submitted filename: Response to Reviewers 2021.docx [file pone.0254658.s008.docx]

12^th^ May, 2021.

Dear Editor,

Thank you for your reply, regarding our Manuscript ‘ PONE-D-21-02655-ISOLATION AND IDENTIFICATION OF MICROORGANISMS ASSOCIATED WITH AUTOMATED TELLER MACHINES ON FEDERAL POLYTECHNIC EDE CAMPUS’

We are so pleased by the elaborate scrutiny of the paper and the corrections needed to make it a stronger and more publishable one.

We have given the manuscript a thorough review and corrected it accordingly; the introductory and the discussion sections have been updated with newer literature and the relevance of the study also highlighted. We have also updated the materials and methods section, as we have included the missing biochemical tests, and needed technical references, also the transfer of swabs to lab has been explained. The results section has been updated to show the relevance of the tables which has also been included in the discussion section. The conclusion and the recommendation sections have been separated and the limitations of the paper added. The manuscript format has been written to reflect the Journals standards, as the SI units has been updated, the Vancouver reference format has been adhered to. The grammatical errors have been somewhat corrected.

Also addressed is the overlapping texts which has been rephrased and the needed references included in the manuscript.

This changes have given the manuscript its needed facelift and I do sincerely hope it can be published with your approval as soon as possible.

Thank you in advance for a most favourable reply.

Yours sincerely,

Dawodu OG PhD
